# Adaptive evolution and co-evolution of chloroplast genomes in Pteridaceae species occupying different habitats: overlapping residues are always highly mutated

**DOI:** 10.1186/s12870-023-04523-1

**Published:** 2023-10-25

**Authors:** Xiaolin Gu, Lingling Li, Sicong Li, Wanxin Shi, Xiaona Zhong, Yingjuan Su, Ting Wang

**Affiliations:** 1 College of Life Sciences, South China Agricultural University, Guangzhou, 510642 China; 2 College of Natural Resources and Environment, South China Agricultural University, Guangzhou, 510642 China; 3 School of Life Sciences, Sun Yat-sen University, Guangzhou, 510275 China; 4 Research Institute of Sun Yat-sen University in Shenzhen, Shenzhen, 518057 China

**Keywords:** Chloroplast, Pteridaceae, Molecular evolution, Intra-molecular co-evolution, Protein tertiary structure

## Abstract

**Background:**

The evolution of protein residues depends on the mutation rates of their encoding nucleotides, but it may also be affected by co-evolution with other residues. Chloroplasts function as environmental sensors, transforming fluctuating environmental signals into different physiological responses. We reasoned that habitat diversity may affect their rate and mode of evolution, which might be evidenced in the chloroplast genome. The Pteridaceae family of ferns occupy an unusually broad range of ecological niches, which provides an ideal system for analysis.

**Results:**

We conducted adaptive evolution and intra-molecular co-evolution analyses of Pteridaceae chloroplast DNAs (cpDNAs). The results indicate that the residues undergoing adaptive evolution and co-evolution were mostly independent, with only a few residues being simultaneously involved in both processes, and these overlapping residues tend to exhibit high mutations. Additionally, our data showed that Pteridaceae chloroplast genes are under purifying selection. Regardless of whether we grouped species by lineage (which corresponded with ecological niches), we determined that positively selected residues mainly target photosynthetic genes.

**Conclusions:**

Our work provides evidence for the adaptive evolution of Pteridaceae cpDNAs, especially photosynthetic genes, to different habitats and sheds light on the adaptive evolution and co-evolution of proteins.

**Supplementary Information:**

The online version contains supplementary material available at 10.1186/s12870-023-04523-1.

## Background

The chloroplast genome of eukaryotes has been substantially reduced due to gene transfer to the host genome and simple gene loss [1]. Therefore, fewer than 5% of genes from the ancestral cyanobacteria remain in chloroplast DNA (cpDNA), mainly those encoding housekeeping and photosynthesis-associated proteins [2]. Nonetheless, chloroplasts are the sites of many important cellular processes, including photosynthesis and lipid, amino acid, chlorophyll, and carotenoid biosynthesis [3, 4]. Moreover, chloroplasts play important roles in plant adaptation to environmental stress, such as drought [5], salinity [6], extreme temperature [7], high light [8], and heavy metal stress [9]. Because chloroplasts act as the energy hubs of plant cells, their homeostasis is readily affected by environmental stress [3, 10]. Chloroplasts function as environmental sensors, transforming fluctuating environmental signals into physiological responses [11–13]. For instance, excessive reactive oxygen species (ROS) in plants can trigger retrograde signaling to subcellular compartments to readjust whole-cell metabolism in order to repair or turn over damaged macromolecules [7, 14]. In addition, the timely expression of chloroplast genes can promote adaptation to environmental fluctuations [15].

Genes encoding proteins with important functions are usually subject to strong evolutionary selection pressure. To measure selection pressure at the molecular level, the nucleotide substitution rate can be used to reflect the changes in protein-coding sequences: non-synonymous substitutions (*dN*) cause amino acid changes, while synonymous substitutions (*dS*) do not [16, 17]. *dN/dS* (ω) is a measure of natural selection that is widely used to detect genes related to environmental adaptation; a ω value greater than 1 is commonly believed to represent positive selection acting on a gene or gene lineage; a ω equal to 1 represents neutral selection, and a ω less than 1 represents purifying selection [18–20]. Many amino acids in functional proteins are conserved due to strong structural and functional constraints; thus, the average *dN* rate is rarely higher than the average *dS* rate [21, 22]. However, some genes may be positively selected under certain environmental conditions, resulting in higher ω values [23].

There may be an evolutionary association between amino acid residues of a given protein: co-evolution may occur between sequences within a protein that physically interact or are functionally relevant, so a change in residues at one site in the molecule may lead to a change in selection pressure at another site. However, amino acid residues located in crucial functional/structural regions will be subject to stronger selection restrictions because these sites may have a huge influence on the protein’s function [24]. Harmful mutations at such sites will be immediately eliminated from the population, but for a mutation that is actively selected for, compensation and substitution may occur to restore any fitness loss due to the mutation [25]. In other words, amino acid co-evolution or correlated mutation is a phenomenon whereby a deleterious substitution at one position is compensated for by another substitution elsewhere in the protein, so that the structure and function of the protein remain stable [26–29]. Notably, if the adaptive positively selected sites of a protein map to positions important for protein structure or function [30–33], and these sites show co-evolutionary relationships, this may indicate their functional/structural dependence [34]. Therefore, identifying the strategies employed by plants for adapting to different environments at the molecular level, along with their co-evolutionary dynamics, can shed light on the evolutionary pattern of species as well as the complex co-adaptation between residues in proteins.

Photosynthesis is particularly sensitive to environmental conditions and plays an important role in the unique niche occupied by land plants [35, 36]. Despite the wide variations in plant morphology and habitat throughout the plant kingdom, the structure and function of most cpDNA-encoded proteins are relatively well conserved. This conservation is largely believed to be driven by strong selection pressure related to the functional requirements of the proteins involved in photosynthesis [37]. Some functional genes with key roles in photosynthesis have undergone adaptive evolution with the “radiation”-type differentiation of species. For instance, genes encoding Rubisco components evolved under positive selection in most terrestrial plant lineages [38], and adaptive evolution of *rbcL* has also been observed in some aquatic plant lineages [39, 40], suggesting that RbcL subunits may have undergone continuous “fine-tuning” in different ecosystems. Similarly, *atpB*, *psaB*, and *rbcL* in *Chlamydomonas* sp. ICE-L show conserved adaptive evolution in extreme environments, which supports the notion that the adaptation of algae to extreme environments is related to the mode of selection of plastid proteins [41].

The Pteridaceae family of ferns contains five subfamilies, 53 genera, and an estimated 1,211 species, contributing to ~ 10% of extant leptosporangiate fern diversity [42, 43]. Pteridaceae have a diverse habitat distribution, ranging from wet tropical to arid regions, and they occupy an unusually broad range of ecological niches [44–46]. Most Pteridaceae species are terrestrial and inhabit open, often rocky environments, but representatives of some *Adiantum* and *Pteris* species are frequent in forests [47]. The Parkerioideae subfamily, represented by *Ceratopteris*, thrives in aquatic habitats, and *Acrostichum* is often associated with mangroves in the intertidal zone [48]. Species in the Cheilanthoideae subfamily are significant components of arid terrestrial habitats [49]. Vitarioid ferns, as sisters to the genus *Adiantum* L., are highly simplified and predominantly epiphytic species [50]. This provides an ideal system for studying the adaptive evolution and intra-molecular co-evolution of cpDNA in a wide range of niches. Previous studies on Pteridaceae have mainly focused on exploring their phylogenetic relationships based on their DNA sequences; however, few studies have focused on the molecular evolution of this family, especially between species inhabiting different environments. In this study, we divided 41 species of Pteridaceae into the terrestrial clade, the epiphytic clade, and the aquatic clade and analyzed the evolutionary dynamics of cpDNA of different habitat groups using common protein-coding sequence data sets to analyze (1) the molecular mechanism of adaptive evolution of Pteridaceae cpDNAs in different groups and (2) the intra-molecular co-evolution patterns of genes involved in this adaptive evolution.

## Results

### Construction of a phylogenetic tree for adaptive evolution and co-evolution analyses

**Fig. 1:**
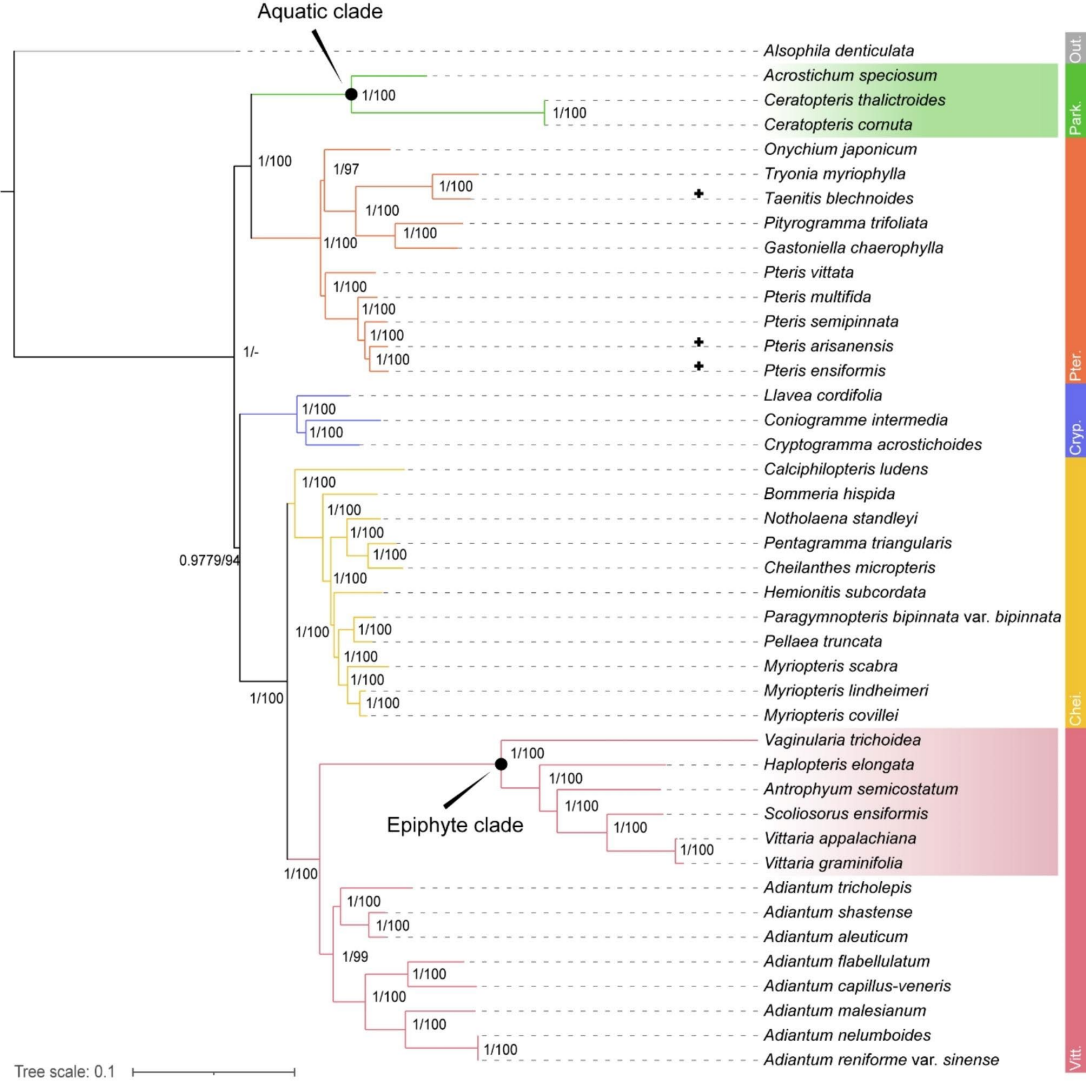
Phylogenetic frame of 41 Pteridaceae species constructed for adaptive evolution and co-evolution analysis. Out.: Outgroup; Park.: Parkerioideae; Pter.: Pteridoideae; Cryp.: Cryptogrammoideae; Chei.: Cheilanthoideae; Vitt.: Vittarioideae. + represents newly added cpDNA samples

We constructed a phylogenetic tree using protein sequences inferred from cpDNAs of 41 Pteridaceae species (Table S1). The topologies and support values from maximum likelihood (ML) and Bayesian inference (BI) analyses were similar, and the 41 species were resolved into five highly supported “subfamily clades”: Parkerioideae, Pteridoideae, Cryptogrammoideae, Cheilanthoideae, and Vittarioideae. *Pteris* and *Adiantum* were each resolved as monophyletic in this analysis, consistent with previous reports [43, 51–53]. We categorized this system into three clades based on their respective habitats: the aquatic clade belonging to the Parkerioideae subfamily, the epiphytic clade consisting of vittarioid ferns, while the terrestrial clade composing of the others (Fig. 1).

### Analysis of selection pressure

We used the basic model to detect the selection pressure range in 41 Pteridaceae species. The ω values of Pteridaceae chloroplast protein-coding genes ranged from 0.0046 to 0.4432 (Table S2), and all 76 common protein-coding genes had ω less than 0.5, indicating that the protein-coding genes of Pteridaceae chloroplasts are dominated by purifying selection.

To test whether the evolutionary pattern of the chloroplast protein-coding genes in the Pteridaceae family is related to adaptability to different habitats, we set the terrestrial, aquatic, or epiphytic ancestral clade as the foreground clade successively when using the branch model and the other two non-target clades as background clade (Specific methods are detailed in Materials and Methods). The branch model will detect positive selection in a lineage only when the average *dN* of all sites is higher than the average *dS* [21]. We discarded five genes with extremely low *dS* values to avoid misestimating the ω value (999): accordingly, we eliminated *infA* from the terrestrial clade, *psbJ* from the epiphytic clade, and *psaI*, *psaJ*, and *psbT* from the aquatic clade. Using the remaining data sets, only when the terrestrial ancestral clade was used as the foreground clade did *rbcL* accept the M2 model, and the ω values became significant after *p*-value correction (ω = 0.0047, *q*-value = 0.0154). When the aquatic ancestral clade or the epiphytic ancestral clade was used as the foreground clade, we observed no significant positive selection after *p*-value correction. No matter which habitat was used as the foreground clade, the test results were mainly ω < 0.5 (Table S3), which also indicates that the Pteridaceae chloroplasts are dominated by purifying selection.

### RELAX analysis

**Fig. 2:**
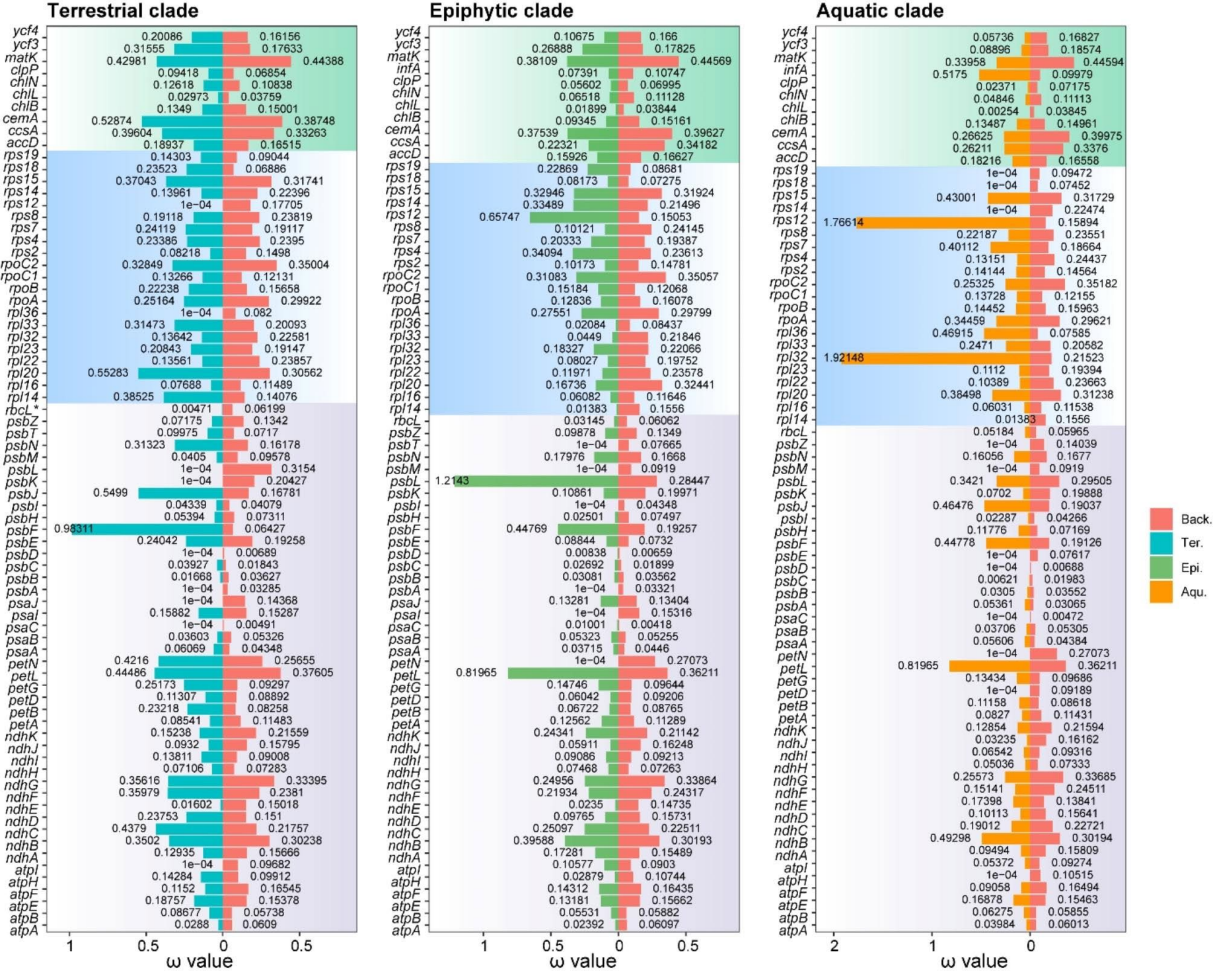
The ω value of each retained protein-coding gene in different foreground clades under the branch model. Purple, blue, and green backgrounds represent genes related to the photosynthetic system, genetic system, and other functions respectively. * marked gene represents significance after *p*-value correction. Back.: Background clade; Ter.: Terrestrial clade; Aqu.: Aquatic clade; Epi.: Epiphytic clade

We identified a few genes with high but not statistically significant ω values in the above data. For instance, the ω values of *psbL* in the epiphytic ancestral clade and *rps12* and *rpl32* in the aquatic ancestral clade were greater than 1 and far higher than their respective background clades (Fig. 2). A higher ω value may be caused by the positive selection of specific genes or the relaxation of natural selection. Based on this result, we tested the selection intensity of Pteridaceae species from different habitats. We determined that the higher ω value of *psbL* in the epiphytic clade may be caused by the relaxation of selection pressure, while those of *rps12* and *rpl32* in the aquatic clade were caused by the intensification of selective pressure. In addition, *rbcL* with significant purifying selection in the terrestrial clade showed significant relaxation of selective pressure (Table S3).

### Analysis of residues under positive selection

As most amino acids in a functional protein are under structural and functional constraints, and adaptive evolution likely affects only a few sites at a few time points [21], we used the site model, which ignores the variation in the ω value between branches, to determine whether any residues have undergone positive selection. Using the Bayes empirical Bayes (BEB) method in the site model with residue reference sites based on *Pteris arisanensis*, we detected positively selected residues within 20 chloroplast protein-coding genes that were statistically significant (Table 1).

Among these genes, *atpE*, *matK*, *ndhF* and *rpoB* rejected the M2a model in the M2a vs. M1a nested model, so we dismissed the results for these genes. Accordingly, 36 positively selected residues were retained in the M2a model (*P* > 95%), which are encoded within 16 genes. Of these genes, 10 are photosynthetic genes (*atpA*, *atpB*, *atpH*, *ndhB*, *ndhK*, *ndhG*, *petB*, *psbF*, *psbL*, and *psbZ*), four are genetic system genes (*rpl16*, *rpoA*, *rpoC2*, and *rps7*), and two are other functional genes (*cemA* and *ycf3*). Furthermore, *atpA atpB* and *ndhK* rejected the M8 model in the M8 vs. M8a nested model, so we dismissed the results for these genes. Accordingly, 59 positively selected residues were retained in the M8 model (*P* > 95%), which are encoded by 17 genes. Among these, nine are photosynthetic genes (*atpE*, *atpH*, *ndhB*, *ndhF*, *ndhG*, *petB*, *psbF*, *psbL*, and *psbZ*), five genetic system genes (*rpl16*, *rpoA*, *rpoB*, *rpoC2*, and *rps7*), and three are other functional genes (*cemA*, *matK*, and *ycf3*). Together, the results of the M2a and M8 models show that most of the positively selected residues are encoded in photosynthetic genes of Pteridaceae.

The branch-site model provided evidence of positive selection on different habitat groups of Pteridaceae. Using the BEB method, we did not detect positively selected residues in the terrestrial ancestral clade. Instead, we identified 3 positively selected residues in the epiphytic ancestral clade and 2 in the aquatic ancestral clade (*P* > 95%, with residue reference sites based on *P. arisanensis*) (Table 1). These residues are encoded by *ndhI* (S50), *psaB* (T511), and *rps19* (R17) in the epiphytic ancestral clade. In the aquatic ancestral clade, they are encoded by *ndhG* (W121) and *rpl33* (T16). These residues showed strong positive selection in the branch-site model, suggesting they may be critical for adaptive evolution in the epiphytic and aquatic ancestral clades of Pteridaceae.

**Table 1:** Parameter estimates for different model selection tests

Gene name	Model	d.f.	2*ΔL*	LRT *P*-value	Positive selection sites (BEB)
**Site Model**					
* atpA*	M2a vs. M1a	2	21.874	0	L308**
	M8 vs. M7	2	113.077	0	Dismiss
	M8 vs. M8a	1	1.5231	0.2172	Not allowed
* atpB*	M2a vs. M1a	2	11.6161	0.0029	H123*, S252**, S474*
	M8 vs. M7	2	111.2862	0	Dismiss
	M8 vs. M8a	1	0.5467	0.4597	Not allowed
* atpE*	M2a vs. M1a	2	0	1	Dismiss
	M8 vs. M7	2	14.7578	0.0006	W20*, L62**
	M8 vs. M8a	1	7.7754	0.0053	Not allowed
* atpH*	M2a vs. M1a	2	6.786	0.0336	L80*
	M8 vs. M7	2	26.0509	0	L57*, P74*, L80**
	M8 vs. M8a	1	5.5447	0.0185	Not allowed
* cemA*	M2a vs. M1a	2	19.1067	0.0001	G152*, R154*, V158*
	M8 vs. M7	2	25.0987	0	G152*, R154**, R155*, V158*, V159*
	M8 vs. M8a	1	14.358	0.0002	Not allowed
* matK*	M2a vs. M1a	2	0	1	Dismiss
	M8 vs. M7	2	37.4691	0	E118*, A346*
	M8 vs. M8a	1	11.667	0.0006	Not allowed
* ndhB*	M2a vs. M1a	2	32.8718	0	P9*, Q68*, T78**, L180*, R209*
	M8 vs. M7	2	58.0883	0	I6*, P9**, T56*, M60*, Q68**, T78**, L180**, L206*, R209**
	M8 vs. M8a	1	17.7176	0	Not allowed
* ndhF*	M2a vs. M1a	2	0	1	Dismiss
	M8 vs. M7	2	51.2991	0	T473*, G516*, L624**, L629*
	M8 vs. M8a	1	11.8531	0.0006	Not allowed
* ndhG*	M2a vs. M1a	2	11.7994	0.0077	K139*
	M8 vs. M7	2	32.2417	0	K139*
	M8 vs. M8a	1	6.2642	0.0091	Not allowed
* ndhK*	M2a vs. M1a	2	15.1025	0.0005	K221**
	M8 vs. M7	2	54.8315	0	Dismiss
	M8 vs. M8a	1	5.3409	0.0919	Not allowed
* petB*	M2a vs. M1a	2	63.9595	0	S2**
	M8 vs. M7	2	81.0342	0	S2**, R118*
	M8 vs. M8a	1	6.6194	0.0101	Not allowed
* psbF*	M2a vs. M1a	2	7.2313	0.0269	S27*
	M8 vs. M7	2	14.4889	0.0007	S27**, S28*
	M8 vs. M8a	1	8.3591	0.0038	Not allowed
* psbL*	M2a vs. M1a	2	18.1735	0.0001	W20**, L24**, S31*
	M8 vs. M7	2	23.78	0	L13*, W20**, L24**, S31**
	M8 vs. M8a	1	18.1734	0	Not allowed
* psbZ*	M2a vs. M1a	2	6.885	0.032	S35*
	M8 vs. M7	2	23.5259	0	S35**
	M8 vs. M8a	1	8.7711	0.0031	Not allowed
* rpl16*	M2a vs. M1a	2	60.897	0	I2**
	M8 vs. M7	2	102.3139	0	I2**
	M8 vs. M8a	1	43.9429	0	Not allowed
* rpoA*	M2a vs. M1a	2	66.9924	0	Q106*, R128*, S173**, D233**, C236*, P241*, Q245*, P249*, F272*
	M8 vs. M7	2	92.6536	0	Q106*, R128*, S147*, S173**, D233**, C236*, P241**, Q245*, P249*, F272*
	M8 vs. M8a	1	61.6783	0	Not allowed
* rpoB*	M2a vs. M1a	2	0	1	Dismiss
	M8 vs. M7	2	93.4487	0	R575*, V597*, E598*, R604**, S606**, P628*
	M8 vs. M8a	1	13.6511	0.0002	Not allowed
* rpoC2*	M2a vs. M1a	2	28.6437	0	F1020*, P1074**
	M8 vs. M7	2	67.1979	0	F1020*, P1074**
	M8 vs. M8a	1	29.8592	0	Not allowed
* rps7*	M2a vs. M1a	2	14.8195	0.0006	S94**, S115*
	M8 vs. M7	2	25.8359	0	S94**, S115*
	M8 vs. M8a	1	15.2237	0.0001	Not allowed
* ycf3*	M2a vs. M1a	2	12.2851	0.0021	P55**
	M8 vs. M7	2	29.1111	0	L75*, Q125*, P55**
	M8 vs. M8a	1	10.7695	0.001	Not allowed
**Branch-site Model**					
**Epiphytic clade**					
* ndhI*	Model A vs. Model A null	1	5.1717	0.023	S50**
* psaB*	Model A vs. Model A null	1	8.405	0.0037	T511*
* rps19*	Model A vs. Model A null	1	5.5583	0.0184	R17**
**Aquatic clade**					
* ndhG*	Model A vs. Model A null	1	10.0159	0.0016	W121*
* rpl33*	Model A vs. Model A null	1	4.2567	0.0391	T16*

d.f.: difference in the number of parameters in the ω distribution; LnL: log-likelihood value; LRT: likelihood ratio test; BEB: Bayes empirical Bayes analysis; * *P* > 95%; ** *P* > 99%.

### Intra-molecular co-evolution analysis

The evolution of residues depends on their intrinsic nucleotide mutation rates and the effects imposed by their complex co-evolutionary networks [24]. Co-evolution dynamics can highlight the intricate co-adaptive relationships between residues in a protein under an estimated timescale [34]. Using the protein sequences of *P. arisanensis* as a reference, we conducted co-evolution analysis of the genes encoding positively selected residues (i.e., adaptively evolving sites) identified using the site model (17 of the 20 protein sequences tested, except for PsbF, PsbL, and PsbZ). The 17 remaining protein sequences (AtpA, AtpB, AtpE, AtpH, CemA, MatK, NdhB, NdhF, NdhG, NdhK, PetB, Rpl16, RpoA, RpoB, RpoC2, Rps7, and Ycf3) contained co-evolved residue pairs (Table 2 displays proteins containing overlapping residues in the co-evolution and adaptive evolution analyses; other co-evolved residue pairs are shown in Table S4). Among these, RpoC2 had the most co-evolved residue pairs (363 pairs), followed by NdhB (90 pairs), AtpB (74), RpoB (50), NdhF (45), CemA (43), AtpA (38), RpoA (37), MatK (33), PetB (20), NdhG (6), NdhK (5), Rpl16 (5), AtpE (4), Ycf3 (4), AtpH (1), and Rps7 (1).

In complex co-evolution networks, some residues frequently appear in different co-evolved pairs. In particular, evolutionary dependence is identified between sites belonging to different domains, which plays an important role in the formation of residue co-evolution networks. For instance, based on the co-evolved pairs (S125 & S252 and S252 & P461) in the AtpB protein sequence, although S125 and P461 do not show direct co-evolution, there may be some indirect co-evolution pressure.

**Table 2:** Information about intra-molecular co-evolved residue pairs for protein sequences containing overlapping residues in co-evolution and adaptive evolution analyses

Co-evolution pairs	Distance (*Å*)	Co-evolution pairs	Distance (*Å*)	Co-evolution pairs	Distance (*Å*)
AtpA					
S57 & E58	3.7	S104 & L247	11.0	L186 & S338	27.8
L68 & S130	20.6	S104 & T258	18.9	N192 & M220	13.9
L68 & N192	44.4	S104 & M315	18.9	E216 & **L308**	36.0
L68 & M220	41.7	S130 & P134	9.5	M220 & L279	38.5
P77 & D103	28.8	S130 & E216	32.4	M220 & R280	41.4
L82 & L186	40.1	S130 & Y294	17.8	L247 & T258	9.6
L82 & M220	35.4	P134 & M220	28.8	L247 & M315	10.9
T83 & K255	43.8	P134 & L279	24.8	K255 & T258	9.6
T83 & T258	38.8	L186 & K189	6.6	K255 & M315	10.0
T83 & M315	39.4	L186 & M220	8.8	T258 & M315	4.5
K95 & L186	36.2	L186 & K255	21.2	L265 & S338	13.7
K95 & L265	26.7	L186 & Q256	17.5	L279 & Y294	17.5
K95 & S338	33.3	L186 & L265	21.1		
AtpB					
K16 & M214	45.5	K50 & **H123**	27.7	S125 & **S252**	17.4
K16 & F472	70.2	K50 & S249	25.8	S125 & P450	44.9
N17 & P35	11.1	E56 & S97	18.5	S125 & Y473	51.8
N17 & E56	18.5	E56 & N124	22.3	S125 & **S474**	48.7
N17 & G88	11.4	E56 & S125	24.7	T126 & S249	16.7
N17 & N124	26.3	E56 & T126	23.9	S183 & M214	12.7
N17 & S197	35.0	E56 & I220	30.8	S183 & S249	21.6
N17 & G301	32.2	E56 & G301	35.6	S183 & P450	18.5
N17 & **S474**	62.6	E56 & N466	71.5	S183 & T490	25.4
N17 & T490	69.0	E56 & **S474**	53.8	S197 & **S252**	9.3
V18 & **H123**	29.3	T77 & **S474**	44.8	S197 & **S474**	28.8
V18 & G492	71.5	T77 & S487	55.3	M214 & S249	22.6
Y20 & A136	39.4	G88 & T490	67.3	M214 & Y473	29.4
F33 & M214	46.5	S97 & L157	41.0	I225 & S249	28.0
F33 & S249	27.2	E102 & S103	3.8	I225 & **S252**	24.8
P35 & E56	27.7	E102 & F109	13.2	S249 & P450	37.5
P35 & N124	36.3	F109 & K263	22.5	**S252** & P461	46.4
P35 & G301	32.6	**H123** & S183	29.9	G301 & **S474**	40.2
P35 & **S474**	68.0	**H123** & K224	13.0	L373 & L406	35.7
N40 & M214	40.2	**H123 & S252**	19.1	L406 & **S474**	30.6
N40 & Y473	58.4	**H123** & G492	54.8	P450 & **S474**	18.4
V46 & E56	10.5	N124 & G301	33.6	Y473 & **S474**	3.8
V47 & E56	8.8	N124 & **S474**	46.5	**S474** & A486	10.2
V47 & S133	27.6	S125 & I225	18.3	T490 & G492	5.3
V47 & N466	72.2	S125 & S249	19.1		
AtpH					
I11 & **L80**	12.8				
MatK					
**E118** & P321	32.2	D204 & S326	25.2	I281 & F359	27.7
Q127 & V146	26.8	I215 & I271	20.7	V291 & K303	19.4
Q127 & L151	28.1	Y252 & E318	16.5	V291 & R383	42.7
Q127 & C433	36.7	Y252 & R482	62.4	R292 & I484	62.0
V146 & C433	38.1	I261 & Y289	21.0	F302 & H305	5.4
V146 & R482	51.4	D262 & Q267	6.9	E318 & R482	54.4
K148 & V337	19.7	Y264 & H307	40.8	P321 & N465	30.8
Y150 & H421	27.2	G274 & K362	35.4	K370 & R449	31.2
L151 & C433	37.8	Y276 & L480	43.8	A403 & T479	12.1
L151 & R449	52.4	K279 & R424	29.0	R424 & T479	23.9
F187 & V337	46.1	K279 & T479	42.8	Y427 & I431	6.5
NdhF					
V221 & L235	21.4	R340 & L433	21.7	S479 & T541	48.0
I224 & L441	37.3	V347 & I609	23.7	S488 & S693	88.0
F225 & V687	44.2	V347 & S676	50.1	V504 & T636	39.2
A226 & V293	13.9	V363 & G608	24.4	S519 & Y659	46.6
L233 & E528	60.5	V363 & F686	65.2	A551 & L604	21.6
L233 & L556	33.8	S366 & L556	33.4	L556 & G608	25.0
L233 & G608	36.4	S366 & I609	26.5	L556 & I609	23.9
L233 & I609	38.3	S366 & T648	30.6	L556 & S676	62.2
A239 & F382	31.2	R377 & F508	42.4	D560 & F678	65.8
F244 & L343	20.2	L403 & V545	15.4	G608 & I609	3.8
I277 & Y351	20.6	E419 & H512	70.5	G608 & F686	69.3
I286 & S693	65.2	E423 & V709	104.6	I609 & S676	57.4
M291 & F678	43.7	G437 & D560	23.5	**L629** & F655	33.1
I310 & S710	95.1	G437 & F678	53.1	L642 & G704	83.3
G318 & F447	11.7	I449 & G685	61.9	Y658 & V683	36.0
PetB					
M1 & R43	47.5	H58 & I161	31.7	L95 & S164	32.3
**S2** & H58	60.3	H58 & P200	30.1	**R118** & M199	11.4
R43 & I161	35.3	H58 & L204	35.9	G125 & P163	25.1
L45 & L95	15.2	T63 & L95	33.3	L160 & S164	8.5
L45 & I161	35.4	T63 & L204	43.1	P163 & M199	31.0
H58 & T63	8.7	L95 & M96	3.8	L165 & L204	32.5
H58 & L95	28.5	L95 & I161	32.4		
RpoB					
**R575** & K609	19.9	L651 & M1049	60.4	R771 & D934	67.9
Y581 & K609	17.3	K653 & S723	44.8	L775 & 4 E105	48.2
E583 & G937	55.4	K653 & N920	29.9	K776 & P939	56.0
G584 & R771	45.1	N674 & T1058	64.4	I779 & Q837	35.4
G584 & D934	50.4	F686 & L728	17.5	T797 & I820	44.1
E594 & T1034	89.6	F686 & T797	31.9	V803 & 7 I106	69.2
V595 & D796	45.7	Q708 & K813	36.4	Y807 & 4 T103	48.3
L631 & G937	56.4	Q708 & E876	62.3	K813 & E876	27.6
L631 & P1020	74.1	Q708 & I1070	72.5	K813 & 0 I107	75.1
F632 & N674	41.1	L728 & T797	24.1	R836 & 7 I106	82.5
E636 & A642	17.5	L728 & P1020	43.8	Q837 & T880	28.6
E636 & Y807	33.2	Q745 & L759	20.3	Q837 & 9 M104	72.5
I637 & D934	50.1	A748 & D800	26.9	E876 & 0 I107	92.9
A642 & V803	30.3	S749 & F1057	36.9	T880 & 9 M104	79.5
A645 & A697	50.0	L758 & R782	40.0	A909 & P914	11.7
L651 & Q837	28.6	R771 & D800	20.3	L993 & 1 E106	31.1
L651 & T880	19.9	R771 & V885	70.9		

Distance: distance between the two alpha carbon atoms. Bold font represents overlapping residues identified by co-evolution and adaptive evolution analyses.

### Relationship between adaptive evolution and co-evolution

**Fig. 3:**
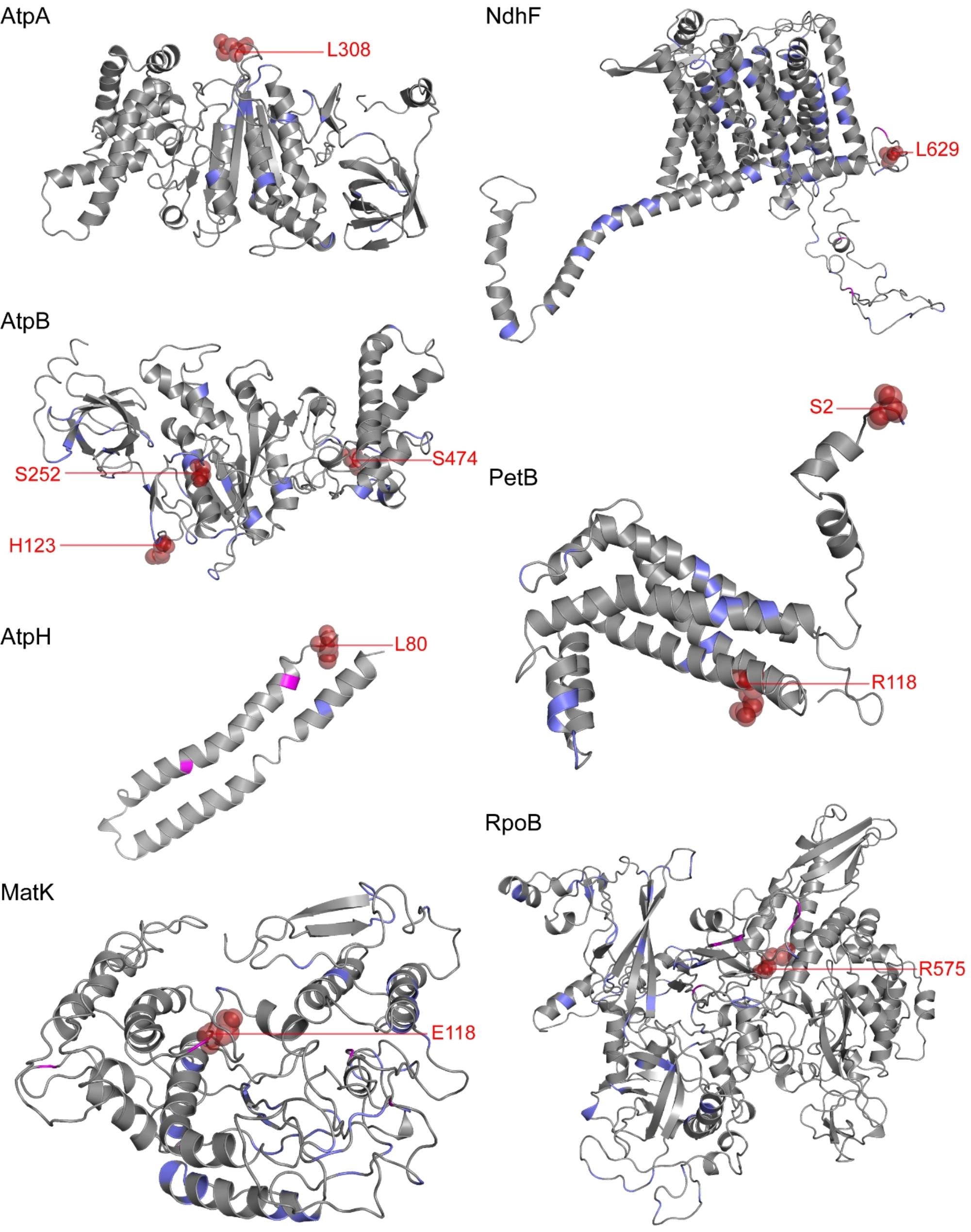
Distribution of positively selected residues and co-evolved residues in the AtpA, AtpB, AtpH, MatK, NdhF, PetB, and RpoB proteins of Pteridaceae. All protein tertiary structures were predicted by homology based on *P. arisanensis*. Purple represents co-evolution residues, magenta represents adaptive evolution residues, and firebrick red represents both and is labeled accordingly

**Fig. 4:**
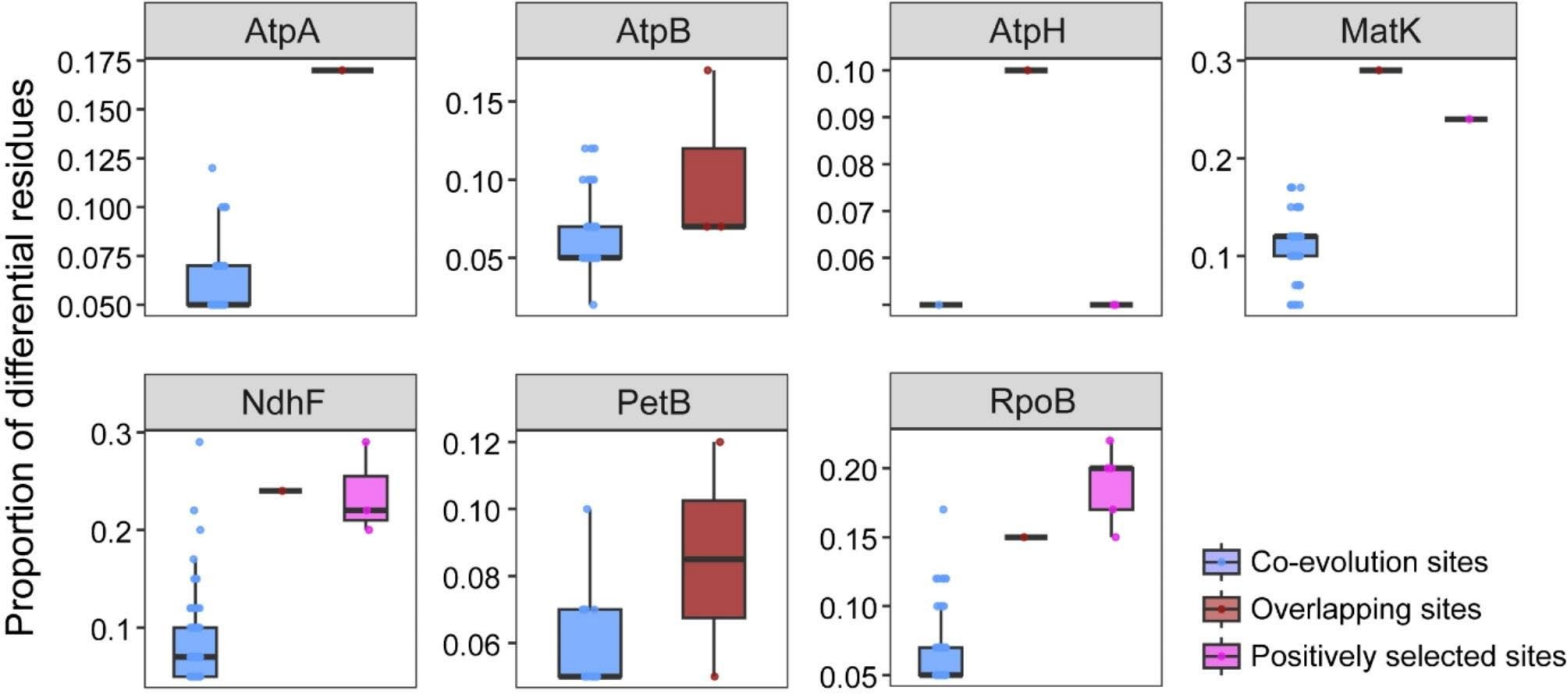
The proportion of differential residues in multiple sequence alignment of co-evolved sites, positively selected sites, and overlapping sites between the two

Having more positively selected residues makes it easier to observe the relationship between positively selected residues and co-evolved residues. Therefore, we used the union of the M2a and M8 model results in the following analyses. We analyzed the relationship between positively selected residues and co-evolved residues by predicting the tertiary structures of protein sequences. The co-evolved residues were scattered throughout the protein sequences (Fig. 3), and some sites were not in a direct co-evolutionary relationship even if they had a relatively close linear distance in the primary structure. For example, in the AtpA protein sequence, the co-evolved residues L82 and T83 were both predicted to interact with the distal sites in the tertiary structure (L82 & M220, distance: 35.4 *Å*; T83 & K255, distance: 43.8 *Å*) (Table 2). In addition, the positively selected residues and co-evolved residues within the protein sequence were mostly independent of each other, with only a few overlapping sites: AtpA (L308), AtpB (H123, S252, and S474), AtpH (L80), MatK (E118), NdhF (L629), PetB (S2 and R118), and RpoB (R575) (Fig. 3). After visualizing the co-evolved residues using Jalview [54], we determined that the co-evolved residues that overlapped with positively selected residues tended to be highly mutated, while the non-overlapping co-evolved residues had fewer mutations (Fig. 4). In this study, we defined a residue site as a high mutation site when the same residue was present in less than half (The results of multiple sequence alignment for specific co-evolved and positively selected residues can be found in Figure S1 and Figure S2, respectively). We employed a chi-square test to assess the conservativeness of residues in overlapping positions. The results indicated a statistical preference for overlapping residues to be located in positions with a low conservation ( χ^2^: 52.30, *p*-value: 0).

## Discussion

Adaptive evolutionary responses to changing environmental conditions result in accelerated evolution and the functional evolution of specific stress-response proteins that favor improved fitness in the new environment [55–57]. In this study, we observed that all protein-coding genes in the Pteridaceae chloroplast genome had ω < 0.5, indicating that Pteridaceae cpDNA has mainly undergone purifying selection. However, only *rbcL* showed a ω value (0.0047) in the terrestrial ancestral clade significantly different from that in the background clade, indicating that this gene has experienced significant purifying selection. Rubisco is crucial for photosynthesis, as it not only fixes CO_2_ [58, 59] but also improves plant growth performance under stress [60]. Truncations and mutations of the conserved N terminus of RbcL can substantially affect Rubisco activity [61]. RbcL has evolved adaptively in different environments and is thought to have undergone “continuous fine-tuning” in different ecosystems [38–40]. However, in the terrestrial clade of the Pteridaceae, *rbcL* is under significant purifying selection and is in a state of relaxed selection. On the other hand, the branch-site model did not detect any positively selected residues in the terrestrial ancestral clade. Therefore, we speculate that the ancestors of Pteridaceae likely grew on land and had completed adaptive evolution, so the terrestrial clade of Pteridaceae is now under purifying selection. Under purifying selection, most non-synonymous substitutions will be purified, so *rbcL* exhibits significant purifying selection.

When we applied the likelihood ratio test (LRT) and BEB test without distinguishing between lineages, 20 genes showed residues under positive selection (significant at the 95% level) (Table 1). These residues were mainly encoded within photosynthetic genes, including genes encoding subunits of ATP synthase (*atpA*, *atpB*, *atpE*, and *atpH*), the predominant site of photosynthetic flux control [62]; NADH dehydrogenase (*ndhB*, *ndhF*, *ndhG*, and *ndhK*), which is important for adaptation of the photosynthetic mechanism to abiotic stress [63]; the cytochrome *b6f* complex (*petB*); and photosystem II (*psbF*, *psbL*, and *psbZ*). These genes encode indispensable components of photosynthesis, and the finding that they are under positive selection indicates that photosynthetic genes play key roles in the adaptive evolution of Pteridaceae. The results of the site model also revealed positively selected genes related to plastid-encoded RNA polymerase (*rpoA*, *rpoB*, and *rpoC2*), which are involved in the transcription of photosynthesis-related genes [64, 65]; and small and large ribosome subunits (*rpl16* and *rps7*). The other functional genes showing positive selection were *cemA*, *matK*, and *ycf3*.

To further elucidate the evolutionary pattern of Pteridaceae chloroplast-encoded proteins, we conducted a co-evolution analysis of the protein sequences encoded by 20 genes including positively selected residues, finding that 17 showed intra-molecular co-evolution. Among these, AtpB, NdhB, RpoB, and RpoC2 encoded abundant co-evolved residues (over 50 co-evolved pairs) and formed a complex co-evolution network (Figure S3); this complexity may be due to their large size, as larger proteins have more residue interactions and more possibilities for co-evolution [25]. The co-evolution approach emphasizes the role of spatial structure information in the study of protein evolution and provides strong evidence to reveal the structural and functional correlation of residue sites [66]. Co-evolved residue pairs often correspond to spatially proximal residues in protein structures [67]. In this study, in addition to the co-evolved pairs with relatively close linear and structural distances, we also observed many co-evolutionary relationships between distal residues at the tertiary structure level, which far outnumbered those of proximal residues (Table 2), suggesting that these co-evolved pairs may be more prone to functional co-evolution [68].

When a species is faced with an extreme environment, some residues within proteins undergo adaptive changes [30–33]. To maintain protein stability, this effect may be mitigated by compensatory changes in other amino acid residues [25]. Therefore, the adaptive evolution of residues in a protein and the intra-molecular co-evolution will tend to be linked. However, our results were contrary to this expectation: residues undergoing adaptive evolution and co-evolution are scattered in the three-dimensional structure of proteins, and their overlap is limited (overlapping residues/adaptively evolving residues: 10/64; overlapping residues/co-evolved residues: 10/741), indicating that the positions of these two categories of sites in the protein sequence are nearly independent. After visualizing multiple amino acid sequences, whereas adaptively evolving residues generally exhibit lower conservation (Figure S2), co-evolved residues tend to display relatively higher conservation (Figure S1). Interestingly, the co-evolved residues that overlap with adaptively evolving residues generally show low conservation: that is, the sequences of the overlapping sites were always highly mutated. On the one hand, non-synonymous substitutions are more strongly affected by natural selection than are synonymous substitutions [69]. If adaptive evolution leads to an increase in the non-synonymous substitution rates, it may indicate that the species is actively adapting to the new environment. Therefore, adaptive evolution is expected to increase the rate of non-synonymous substitutions. Notably, functionally/structurally important residues are often subject to strong selection constraints and thus tend to be conserved, even these are subject to mutation under extreme environmental stress. On the other hand, because the structure and function of a macromolecule depend on complex interactions between residues, such changes can alter crucial interactions with other residues. Thus, the fitness effect of a mutation at a given location may depend on the state of interacting residues, resulting in non-independent evolution (co-evolution) [70]. This co-evolution should generally be quite slow, with these residues showing greater conservation [25]. Moreover, the geometry of interactions in the tertiary structures of proteins may vary in time and sequence, with the residues involved potentially changing over time during the evolutionary history of a molecule [25]. Thus, co-evolved residue pairs and adaptively evolved residue sites may vary in time and space. Therefore, one possible explanation for the presence of these overlapping residues is that adaptive evolution and intra-molecular co-evolution in species may be driven by distinct mechanisms, and they happen to overlap. Notably, these overlapping residues tend to exhibit lower conservation.

We also detected genes encoding positively selected residues in the epiphytic and the aquatic clades (95% level, BEB method): *ndhG* (W121) and *rpl33* (T16) in the aquatic ancestral clade and *ndhI* (S50), *psaB* (T511), and *rps19* (R17) in the epiphytic ancestral clade. The habitats of epiphytic species in a tree canopy are diverse, with some limited to smaller branches and others to larger branches or trunks [71]. The aquatic *A. speciosum* usually grows in the shady understory of a mangrove forest and is frequently flooded by tides [72], while *Ceratopteris* species are often restricted to aquatic habitats, such as those near ponds and streams [73]. Such shade-living species are especially vulnerable to damage to the photosynthetic machinery due to excessive light. The genes that were positively selected in these clades encode proteins crucial to this machinery: NdhG, NdhI, and PsaB are involved in photosystem I (PSI) cyclic electron transport [74–76], and the NDH complex appears to be particularly important for enabling the photosynthetic machinery to adapt to stress conditions [63] and/or alleviating stress [75]. Rpl33 and Rps19 are ribosomal proteins, with Rpl33 being necessary to maintain sufficient cpDNA translation under cold conditions [77]. However, we did not detect positively selected residues in the terrestrial ancestral clade, possibly because the Pteridaceae are descended from a terrestrial ancestor that was previously adapted to such environments. In summary, some residues encoded within Pteridaceae chloroplast genes showed positive selection, indicating that they play an important role in the adaptive evolution to different habitats.

## Conclusion

Pteridaceae cpDNAs have mainly undergone purifying selection. Only *rbcL* in the terrestrial clade showed significant purification selection and is in a state of relaxed selection. Regardless of whether the lineage was distinguished, the positively selected residues were mostly encoded by photosynthetic genes, indicating that photosynthetic genes play an important role in the adaptive evolution of Pteridaceae species. We carried out a co-evolution analysis of 20 genes encoding adaptively evolved residues to explore the complex evolutionary pattern of proteins. These adaptively evolved sites and co-evolved sites were mostly independent, with only a few overlapping sites, and the amino acid sequences of these overlapping sites were always highly mutated. These overlapping sites may be due to the different mechanisms between adaptive evolution and co-evolution, and they may overlap in different spaces and times. Obtaining more structural/functional information for these overlapping sites will be crucial for a deeper understanding of the relationship between adaptive evolution and co-evolution. Here, we present evidence at the molecular level about the adaptive evolution of Pteridaceae cpDNAs to different habitats and provide insight into the adaptive evolution and co-evolution of proteins.

## Materials and methods

### Sample collection

Fresh leaves of *Pteris ensiformis*, *P. arisanensis*, and *Taenitis blechnoides* were sampled from the campus of Shenzhen Fairy Lake Botanical Garden (location: E114°09′, N22°34′; altitude: 944 m), quickly frozen in liquid nitrogen, and stored at − 80 °C until use. The plant materials used in the study were identified by Ting Wang, and specimens were stored in the Herbarium of the College of Life Sciences, South China Agricultural University (Specimen numbers of *P. ensiformis*, *P. arisanensis*, and *T. blechnoides* are GXL20210903, GXL20210904 and GXL20210905, respectively).

### Library preparation, sequencing and genome assembly

DNA was extracted from the samples using a Tiangen Plant Genome DNA Kit (Tiangen Biotech Co., Ltd., Beijing, China) according to the manufacturer’s instructions. The Illumina NovaSeq6000 platform was used for sequencing. The complete chloroplast genomes of *P. arisanensis* and *T. blechnoides* were assembled using GetOrganelle [78]. However, the complete chloroplast genome of *P. ensiformis* failed to assemble into a circle using GetOrganelle and was instead assembled using Novoplasty [79]. The sequences were submitted to the National Center for Biotechnology Information (NCBI) under GenBank accession numbers OP441371 (*P. arisanensis*), OP743918 (*P. ensiformis*), and OP743919 (*T. blechnoides*).

### DNA sequence alignment and phylogenetic analysis

The complete chloroplast genomes of 38 Pteridaceae species and *Alsophila denticulata* (outgroup) were downloaded from GenBank (Table S1). Combining our three newly sequenced species, a total of 41 Pteridaceae species were examined, covering all subfamilies. Seventy-six common but different protein-coding sequences of these species were retained (Table S1), MAFFT [80] was used to perform sequence alignment, and the gap area was deleted to exclude poorly aligned positions. PhyloSuite [81] was used to concatenate these sequences into a dataset for phylogenetic analysis. The maximum likelihood (ML) tree was inferred using RAxML [82], GTRGAMMAI was selected as the nucleotide substitution model, and bootstrap values for each branch were obtained by performing 1,000 bootstrap replicates. The Bayesian inference (BI) tree was established by MrBayes [83] and was estimated by running 2,000,000 generations (Nst = 6, rates = invgamma).

### Analysis of selection pressure and adaptive evolution

The 76 common non-repeating protein-coding sequences were used to build independent data sets (Table S1). The 41 Pteridaceae species were classified into three clades based on their habitat: the terrestrial, aquatic, and epiphyte clades. Before performing sequence alignment, the stop codon caused by RNA editing inside the sequences was modified, and the tail stop codon was removed. Alignment gaps and uncertainties were deleted to avoid false positives.

Selection pressure was analyzed using the CODEML [16] program. The ω values of each common protein-coding gene were calculated under the basic model (model = 0, Nsites = 0, which assumes no site-wise or branch-wise *dN*/*dS* variation). The branch model was applied by comparing the single-ratio model (M0) and two-ratio model (M2) (using the likelihood ratio test (LRT) with a χ^2^ distribution); the M0 hypothesis was rejected if *P* < 0.05. The M0 model assumes that all Pteridaceae clades have the same ratio of non-synonymous to synonymous substitution rates; the M2 model assumes different ω between the foreground clade and background clade. The false discovery rate (FDR) correction was applied to the *P* values calculated above [84].

Positive selection models (M2a and M8) and null hypothesis models (M1a, M7, and M8a) provided by PAML [16] were used to perform site adaptive evolutionary analyses on a dataset of shared genes from 41 Pteridaceae species. Three sets of nested models (M2a vs. M1a, M8 vs. M7, and M8a vs. M8) were used to infer the genes experiencing positive selection. If the positive selection model significantly outperformed the null hypothesis model (*p* < 0.05), the genes were assumed to be under positive selection, where M8a vs. M8 resulted in lower false positive results.

The branch-site model allows different site-encoding genes to have different values of ω in different branches of the phylogenetic tree in order to test whether positive selection acts on certain sites in the foreground clade. Model A (model = 2, Nsites = 2, fixed omega = 0, omega = 2) assumes that only the foreground clade undergoes positive selection; Model A null (model = 2, Nsites = 2, fixed omega = 1, omega = 1) fixes the ω of the foreground clade in model A to 1. If Model A is significantly better than Model A null (*p* < 0.05), then the gene has undergone positive selection in the foreground clade.

In the above models, the LRT was compared to a χ^2^ null distribution with the corresponding degrees of freedom. All sites under positive selection were retrieved using the Bayes empirical Bayes (BEB) method.

### Natural selection pressure analyses

Parameter *K* was calculated for the data by running RELAX (implemented in HYPHY v2.5.42) [85] to test the relaxation of natural selection, the selection pressure in this context includes both purifying selection and positive selection. The *K* parameter is related to the value of ω as (ω background)^*K*^ = (ω foreground) [85]. *K* < 1 is indicative of relaxed natural selection and *K* > 1 suggests intensification in the test compared to the background. RELAX was used for LRT analysis by comparing the model with *K* = 1 to the model with *K* < 1 (or *K* > 1). If *K* < 1, *P* < 0.05 indicates that the test clade was under significantly relaxed selection, and if *K* > 1, *P* < 0.05 indicates that the test clade was under significantly intensified selection.

### Co-evolution analysis

To further explore adaptive evolution at the molecular level, co-evolution analysis was conducted on the protein sequences encoded by genes that have undergone adaptive evolution in a specific lineage, and the structures of proteins with co-evolved residue pairs were predicted in order to understand their co-evolutionary mechanism. Co-evolution analysis was performed using the program CAPS [24], which reveals structural and functional correlations between sites by detecting whether amino acid site variants are associated. The alpha-value in the program was set to 0.01; the random sampling value was set to 1000. Protein 3D structure was predicted using Phyre2 [86] online in INTENSIVE mode and visualized using PyMOL (http://www.pymol.org). Multiple sequences of co-evolved residues were checked using Jalview [54].

### Electronic supplementary material

Below is the link to the electronic supplementary material.

Supplementary Material 1

Supplementary Material 2

Supplementary Material 3

Supplementary Material 4

## Data Availability

These sequence data have been submitted to the GenBank databases under accession numbers OP441371, OP743918 and OP743919. The data underlying this article are available in the article and in its online supplementary material.
