# Supplementary material for: Adaptive evolution and co-evolution of chloroplast genomes in Pteridaceae species occupying different habitats: overlapping residues are always highly mutated
Source: BMC Plant Biol. 2023 Oct 25;23:511. doi: 10.1186/s12870-023-04523-1 (PMC10598918; doi:10.1186/s12870-023-04523-1)
Supplement: Supplementary file 1 — Supplementary Material 1 [file 12870_2023_4523_MOESM1_ESM.docx]

**Table S1.** The complete cpDNA accession number of plant materials in this study

| **Subfamily** | **Genus** | **Organism Name** | **Accession No.** | **Common Protein-coding Genes (Pteridaceae / All)** |
| --- | --- | --- | --- | --- |
| Cheilanthoideae | *Bommeria* | *B. hispida* | NC_040206.1 | *accD*, *atpA* (NC_061171.1; NC_053768.1), *atpB*, *atpE*, *atpF*, *atpH*, *atpI*, *ccsA*, *cemA*, *chlB* (NC_061171.1), *chlL* (NC_061171.1), *chlN* (NC_061171.1), *clpP*, *infA* (NC_061171.1; NC_053768.1), *matK*, *ndhA*, *ndhB* (NC_057002.1; NC_062137.1), *ndhC*, *ndhD*, *ndhE*, *ndhF* (NC_061171.1; NC_053768.1), *ndhG*, *ndhH*, *ndhI*, *ndhJ*, *ndhK*, *petA* (NC_061171.1; NC_053768.1), *petB* (NC_040215.1), *petD*, *petG*, *petL*, *petN*, *psaA*, *psaB*, *psaC*, *psaI*, *psaJ*, *psbA*, *psbB*, *psbC*, *psbD*, *psbE*, *psbF* (NC_061171.1; NC_053768.1), *psbH*, *psbI*, *psbJ*, *psbK*, *psbL*, *psbM*, *psbN*, *psbT*, *psbZ*, *rbcL*, *rpl14*, *rpl16* (NC_039724.1), *rpl20*, *rpl22*, *rpl23*, *rpl32*, *rpl33*, *rpl36* (NC_053768.1), *rpoA* (NC_061171.1; NC_053768.1), *rpoB* (NC_061171.1; NC_053768.1), *rpoC1* (NC_061171.1; NC_053768.1), *rpoC2*, *rps2* (NC_061171.1; NC_053768.1), *rps4*, *rps7* (NC_061171.1; NC_053768.1), *rps8*, *rps12*, *rps14*, *rps15*, *rps18*, *rps19*, *ycf3*, *ycf4* |
|  | *Calciphilopteris* | *C. ludens* | NC_040214.1 |  |
|  | *Cheilanthes* | *C. micropteris* | NC_040174.1 |  |
|  | *Hemionitis* | *H. subcordata* | NC_040173.1 |  |
|  | *Myriopteris* | *M. covillei* | NC_039724.1 |  |
|  |  | *M. lindheimeri* | NC_014592.1 |  |
|  |  | *M. scabra* | NC_040213.1 |  |
|  | *Notholaena* | *N. standleyi* | NC_040203.1 |  |
|  | *Paragymnopteris* | *P. bipinnata* var. *bipinnata* | NC_061171.1 |  |
|  | *Pellaea* | *P. truncata* | NC_040202.1 |  |
|  | *Pentagramma* | *P. triangularis* | NC_040171.1 |  |
| Cryptogrammoideae | *Coniogramme* | *C. intermedia* | NC_057002.1 |  |
|  | *Cryptogramma* | *C. acrostichoides* | NC_040211.1 |  |
|  | *Llavea* | *L. cordifolia* | NC_040216.1 |  |
| Parkerioideae | *Acrostichum* | *A. speciosum* | NC_053768.1 |  |
|  | *Ceratopteris* | *C. cornuta* | MH173068.1 |  |
|  |  | *C. thalictroides* | NC_062137.1 |  |
| Pteridoideae | *Gastoniella* | *G. chaerophylla* | NC_040210.1 |  |
|  | *Onychium* | *O. japonicum* | NC_040205.1 |  |
|  | *Pityrogramma* | *P. trifoliata* | NC_040207.1 |  |
|  | *Pteris* | *P. arisanensis* | OP441371 |  |
|  |  | *P. ensiformis* | OP743918 |  |
|  |  | *P. multifida* | NC_058883.1 |  |
|  |  | *P. semipinnata* | NC_060734.1 |  |
|  |  | *P. vittata* | MH173082.1 |  |
|  | *Taenitis* | *T. blechnoides* | OP743919 |  |
|  | *Tryonia* | *T. myriophylla* | NC_040208.1 |  |
| Vittarioideae | *Adiantum* | *A. aleuticum* | NC_040209.1 |  |
|  |  | *A. capillus-veneris* | NC_004766.1 |  |
|  |  | *A. flabellulatum* | NC_064144.1 |  |
|  |  | *A. malesianum* | NC_063331.1 |  |
|  |  | *A. nelumboides* | NC_050350.1 |  |
|  |  | *A. reniforme* var. *sinense* | NC_062433.1 |  |
|  |  | *A. shastense* | NC_037478.1 |  |
|  |  | *A. tricholepis* | NC_040172.1 |  |
|  | *Antrophyum* | *A. semicostatum* | NC_040176.1 |  |
|  | *Haplopteris* | *H. elongata* | NC_040215.1 |  |
|  | *Scoliosorus* | *S. ensiformis* | NC_040218.1 |  |
|  | *Vaginularia* | *V. trichoidea* | NC_040175.1 |  |
|  | *Vittaria* | *V. appalachiana* | NC_040219.1 |  |
|  |  | *V. graminifolia* | NC_040217.1 |  |
| Outgroup | *Alsophila* | *A. denticulata* | NC_058591.1 |  |

Whether or not an outgroup is added, the common protein-coding sequences remain consistent. The accession number annotated after the common protein-coding sequences represents a homologous sequence that was supplemented through BLAST for the corresponding species.
