# Supplementary material for: Adaptive evolution and co-evolution of chloroplast genomes in Pteridaceae species occupying different habitats: overlapping residues are always highly mutated
Source: BMC Plant Biol. 2023 Oct 25;23:511. doi: 10.1186/s12870-023-04523-1 (PMC10598918; doi:10.1186/s12870-023-04523-1)
Supplement: Supplementary file 2 — Supplementary Material 2 [file 12870_2023_4523_MOESM2_ESM.docx]

**Table S2.** The *dN/dS* values of the Pteridaceae protein coding genes under the basic model

| **Gene** | ***dN/dS*** | **Gene** | ***dN/dS*** | **Gene** | ***dN/dS*** | **Gene** | ***dN/dS*** |
| --- | --- | --- | --- | --- | --- | --- | --- |
| *accD* | 0.1660 | *ndhE* | 0.1396 | *psbB* | 0.0354 | *rpl23* | 0.1922 |
| *atpA* | 0.0593 | *ndhF* | 0.2424 | *psbC* | 0.0194 | *rpl32* | 0.2191 |
| *atpB* | 0.0587 | *ndhG* | 0.3348 | *psbD* | 0.0067 | *rpl33* | 0.2068 |
| *atpE* | 0.1552 | *ndhH* | 0.0727 | *psbE* | 0.0742 | *rpl36* | 0.0787 |
| *atpF* | 0.1634 | *ndhI* | 0.0921 | *psbF* | 0.1970 | *rpoA* | 0.2971 |
| *atpH* | 0.1007 | *ndhJ* | 0.1556 | *psbH* | 0.0726 | *rpoB* | 0.1593 |
| *atpI* | 0.0911 | *ndhK* | 0.2125 | *psbI* | 0.0409 | *rpoC1* | 0.1219 |
| *ccsA* | 0.3357 | *petA* | 0.1133 | *psbJ* | 0.1955 | *rpoC2* | 0.3490 |
| *cemA* | 0.3955 | *petB* | 0.0868 | *psbK* | 0.1955 | *rps2* | 0.1455 |
| *chlB* | 0.1492 | *petD* | 0.0903 | *psbL* | 0.2961 | *rps4* | 0.2393 |
| *chlL* | 0.0372 | *petG* | 0.0982 | *psbM* | 0.0918 | *rps7* | 0.1941 |
| *chlN* | 0.1092 | *petL* | 0.3797 | *psbN* | 0.1674 | *rps8* | 0.2351 |
| *clpP* | 0.0694 | *petN* | 0.2631 | *psbT* | 0.0747 | *rps12* | 0.1607 |
| *infA* | 0.1060 | *psaA* | 0.0442 | *psbZ* | 0.1334 | *rps14* | 0.2186 |
| *matK* | 0.4432 | *psaB* | 0.0526 | *rbcL* | 0.0595 | *rps15* | 0.3196 |
| *ndhA* | 0.1556 | *psaC* | 0.0046 | *rpl14* | 0.1495 | *rps18* | 0.0733 |
| *ndhB* | 0.3043 | *psaI* | 0.1532 | *rpl16* | 0.1136 | *rps19* | 0.0924 |
| *ndhC* | 0.2261 | *psaJ* | 0.1340 | *rpl20* | 0.3135 | *ycf3* | 0.1821 |
| *ndhD* | 0.1547 | *psbA* | 0.0312 | *rpl22* | 0.2316 | *ycf4* | 0.1635 |
