# Supplementary material for: Adaptive evolution and co-evolution of chloroplast genomes in Pteridaceae species occupying different habitats: overlapping residues are always highly mutated
Source: BMC Plant Biol. 2023 Oct 25;23:511. doi: 10.1186/s12870-023-04523-1 (PMC10598918; doi:10.1186/s12870-023-04523-1)
Supplement: Supplementary file 4 — Supplementary Material 4 [file 12870_2023_4523_MOESM4_ESM.docx]

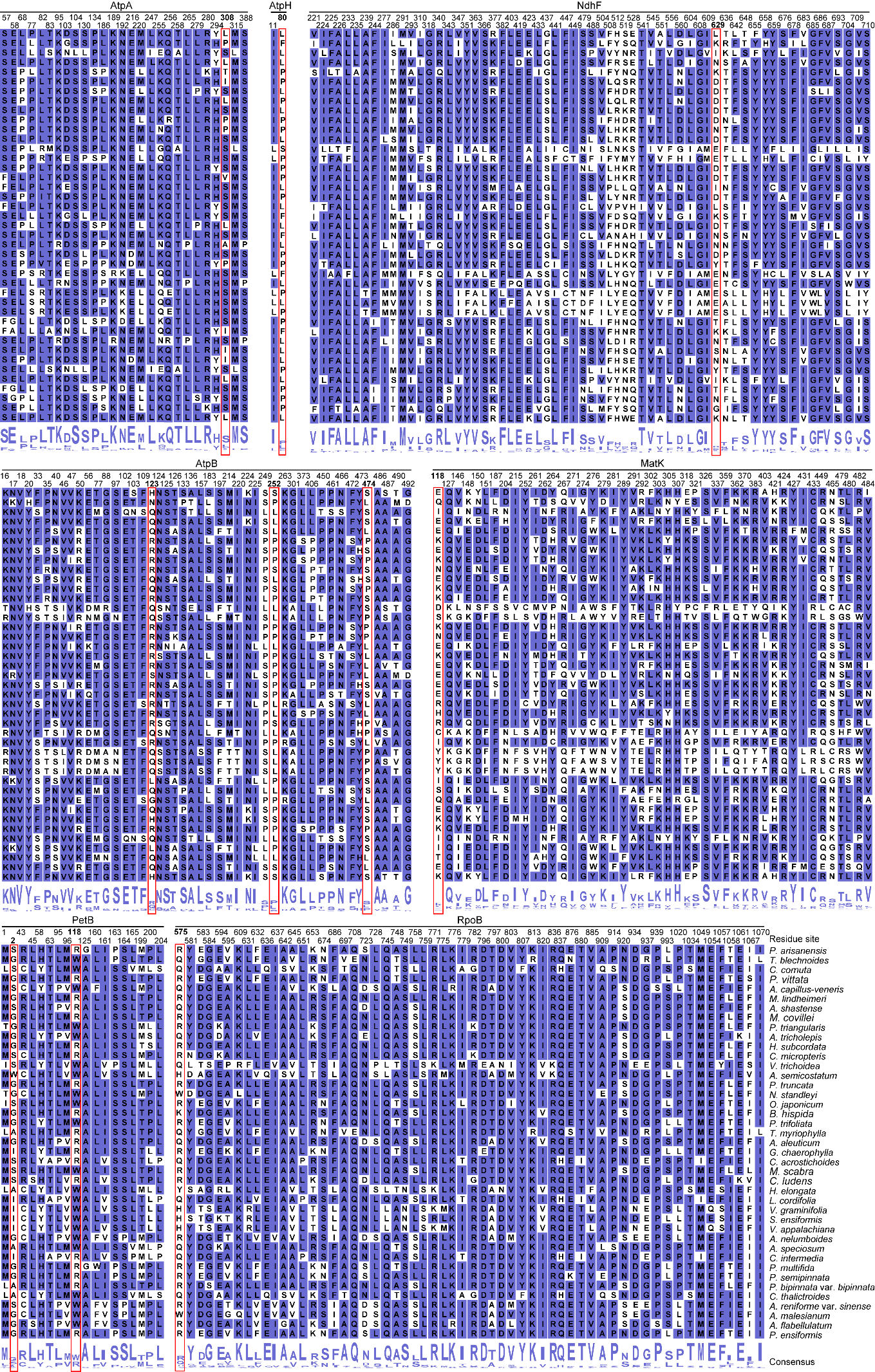


**Figure S1.** Multiple sequence comparison of co-evolved residues. The overlapping sites of co-evolved residues and positively selected residues (*P* > 95%) are highlighted in bold font and enclosed in a red box. At each site, residues in multiple sequences that exceed half will be color-coded for emphasis.


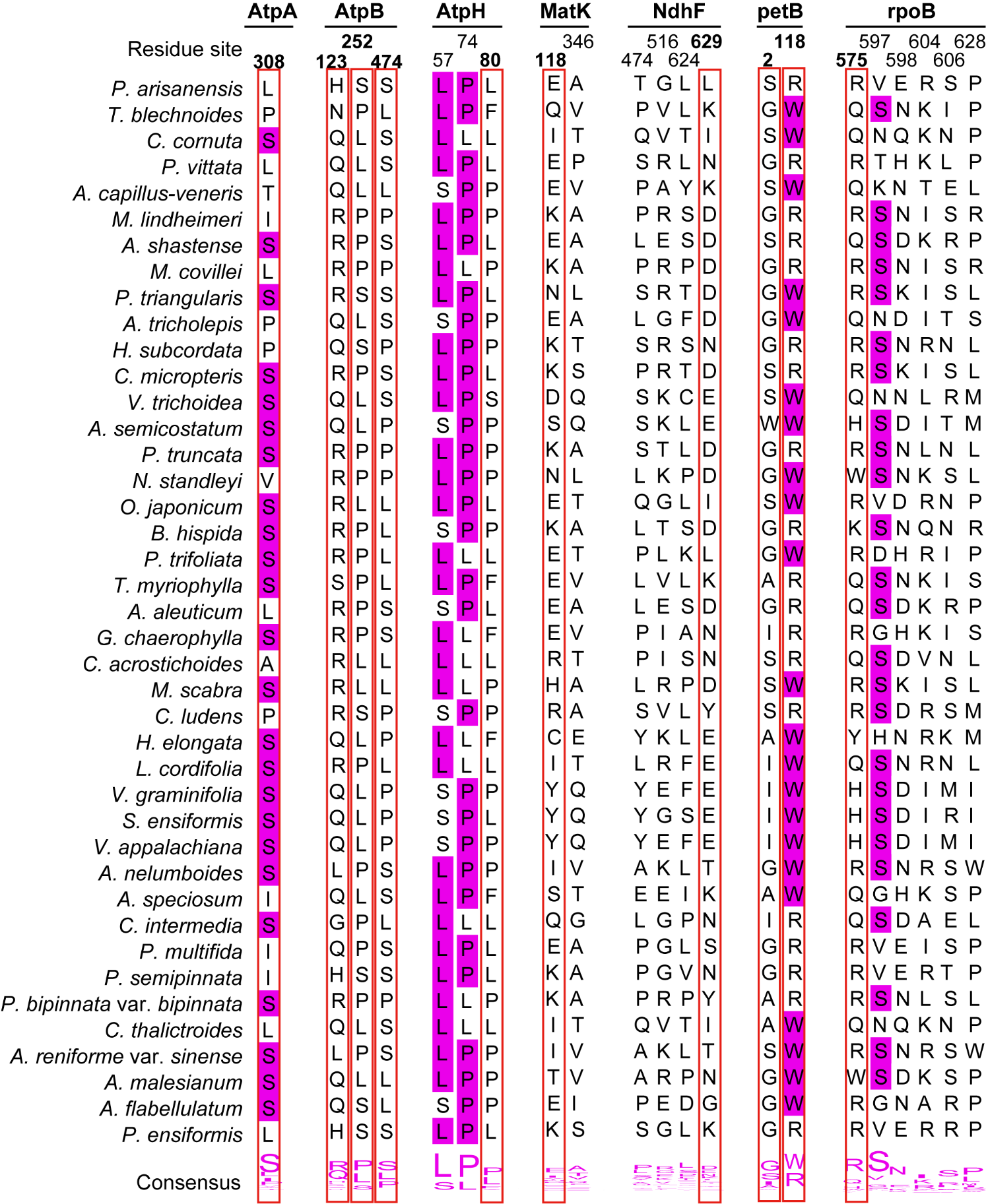


**Figure S2.** Multiple sequence comparison of positively selected residues. The overlapping sites of co-evolved residues and positively selected residues (*P* > 95%) are highlighted in bold font and enclosed in a red box. At each site, residues in multiple sequences that exceed half will be color-coded for emphasis.

**
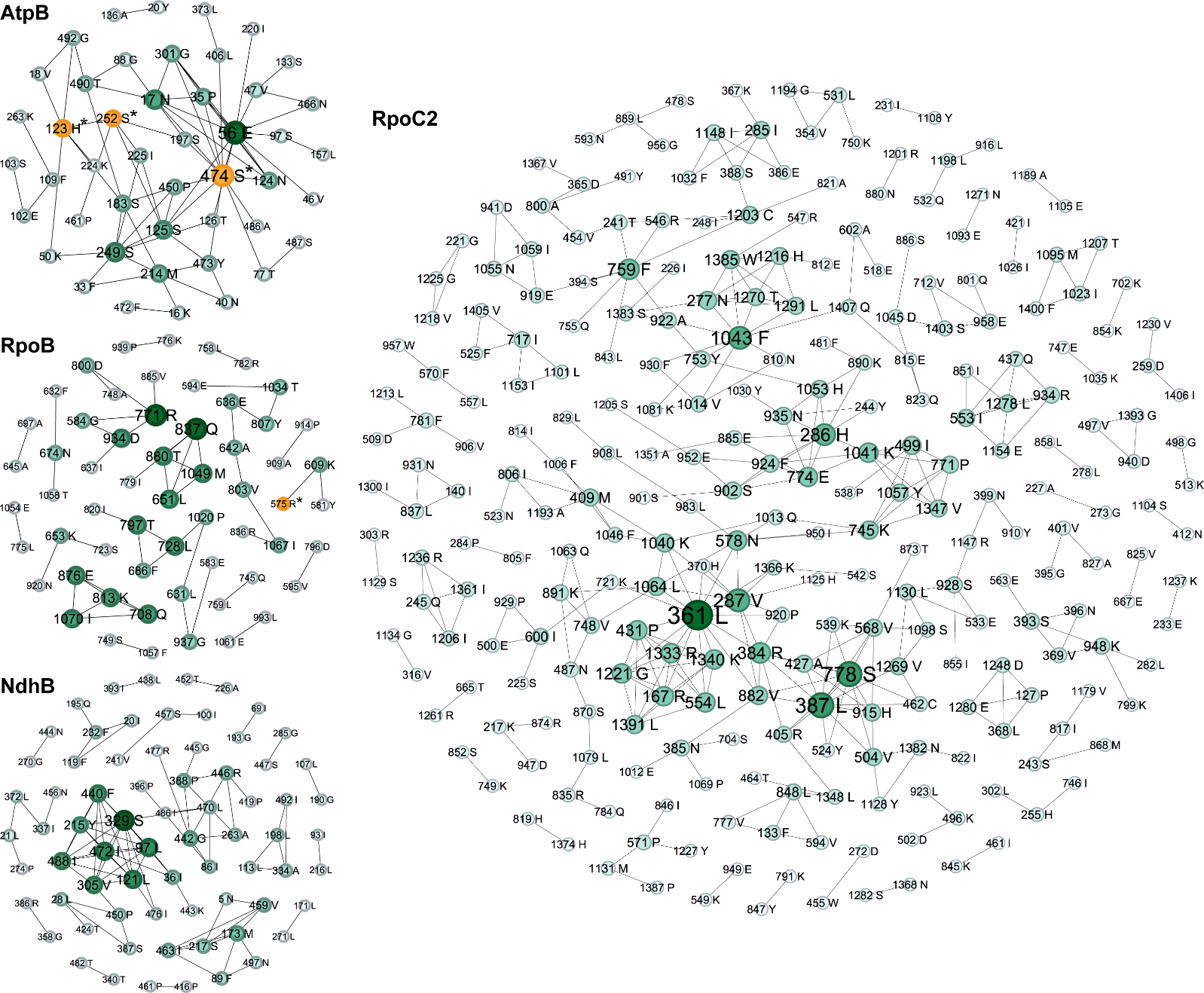
**

**Figure S3.** The intra-molecular co-evolution network of AtpB, NdhB, RpoB and RpoC2 protein sequences. Residues that occur more frequently in co-evolved residue pairs are depicted as darker and larger. Orange residues represent overlap with the positively selected residues. * represents *P* > 95%.
